# Two Human Infections with Diverse Europe-1 Crimean-Congo Hemorrhagic Fever Virus Strains, North Macedonia, 2024

**DOI:** 10.3201/eid3102.241249

**Published:** 2025-02

**Authors:** Dejan Jakimovski, Kostadin Poposki, Marija Dimzova, Marija Cvetanovska, Fadil Cana, Ivana Bogdan, Alejandro Cabezas-Cruz, Brigitta Zana, Zsófia Lanszki, Zsófia Tauber, Tamás Görföl, Krisztián Bányai, Ágota Ábrahám, Pavle Banović, Gábor Kemenesi

**Affiliations:** Saints Cyril and Methodius University in Skopje Faculty of Medicine, Skopje, North Macedonia (D. Jakimovski, K. Poposki, M. Dimzova, M. Cvetanovska); University Clinic for Infectious Diseases and Febrile Conditions, Skopje (D. Jakimovski, K. Poposki, M. Dimzova, M. Cvetanovska, F. Cana); Balkan Association for Vector-Borne Diseases, Novi Sad, Serbia (D. Jakimovski, K. Poposki, I. Bogdan, P. Banović, G. Kemenesi); Pasteur Institute Novi Sad, Novi Sad (I. Bogdan, P. Banović); Laboratoire de Santé Animale, Maisons-Alfort, France (A. Cabezas-Cruz); University of Pécs Szentágothai Research Centre, Pécs, Hungary (B. Zana, Z. Lanszki, Z. Tauber, T. Görföl, K. Bányai, A. Ábrahám, G. Kemenesi); University of Veterinary Medicine, Budapest (K. Bányai); University of Novi Sad Faculty of Medicine, Novi Sad (P. Banović)

**Keywords:** Crimean-Congo hemorrhagic fever, viruses, CCHFV, North Macedonia, phylogenetic analysis, Balkan, vector-borne infections, tickborne, zoonoses, ticks

## Abstract

Until 2023, North Macedonia had not reported a Crimean-Congo hemorrhagic fever (CCHF) case for >50 years. In 2024, increased clinical vigilance identified and characterized 2 novel CCHF cases. Genetic analysis and the identification of possible reassortment indicate North Macedonia as an interaction zone between CCHF virus isolates from Turkey and Kosovo.

Crimean-Congo hemorrhagic fever (CCHF) is a severe zoonotic disease endemic in various regions of Europe, Asia, and Africa, including the Balkans, central Asia, and sub-Saharan Africa (*1*). The disease is caused by the CCHF virus (CCHFV), which is predominantly maintained and transmitted by *Hyalomma* spp. ticks. However, the virus also can be transmitted to humans through direct contact with the bodily fluids of infected animals and humans (*2*). In 2023, CCHF reemerged in North Macedonia (*3*; D. Jakimovski et al., unpub. data, https://doi.org/10.21203/rs.3.rs-4360716/v1), having been absent for >50 years since a 1970 outbreak. The combined mortality rate for the 1970 and 2023 outbreaks was 18.75% (3/16 cases) (*4*; D. Jakimovski et al., unpub. data). In response to this escalating public health concern in the Balkan Region (D. Jakimovski et al., unpub. data), the Balkan Association for Vector-Borne Diseases implemented a strategic plan emphasizing clinical vigilance and capacity sharing. Those efforts culminated in the detection and characterization of an autochthonous CCHFV strain linked to the 2023 outbreak (D. Jakimovski et al., unpub. data). Our study explored the reemergence of CCHF cases in North Macedonia, emphasizing the co-circulation of multiple autochthonous viral strains.

## The Study

**Figure 1:**
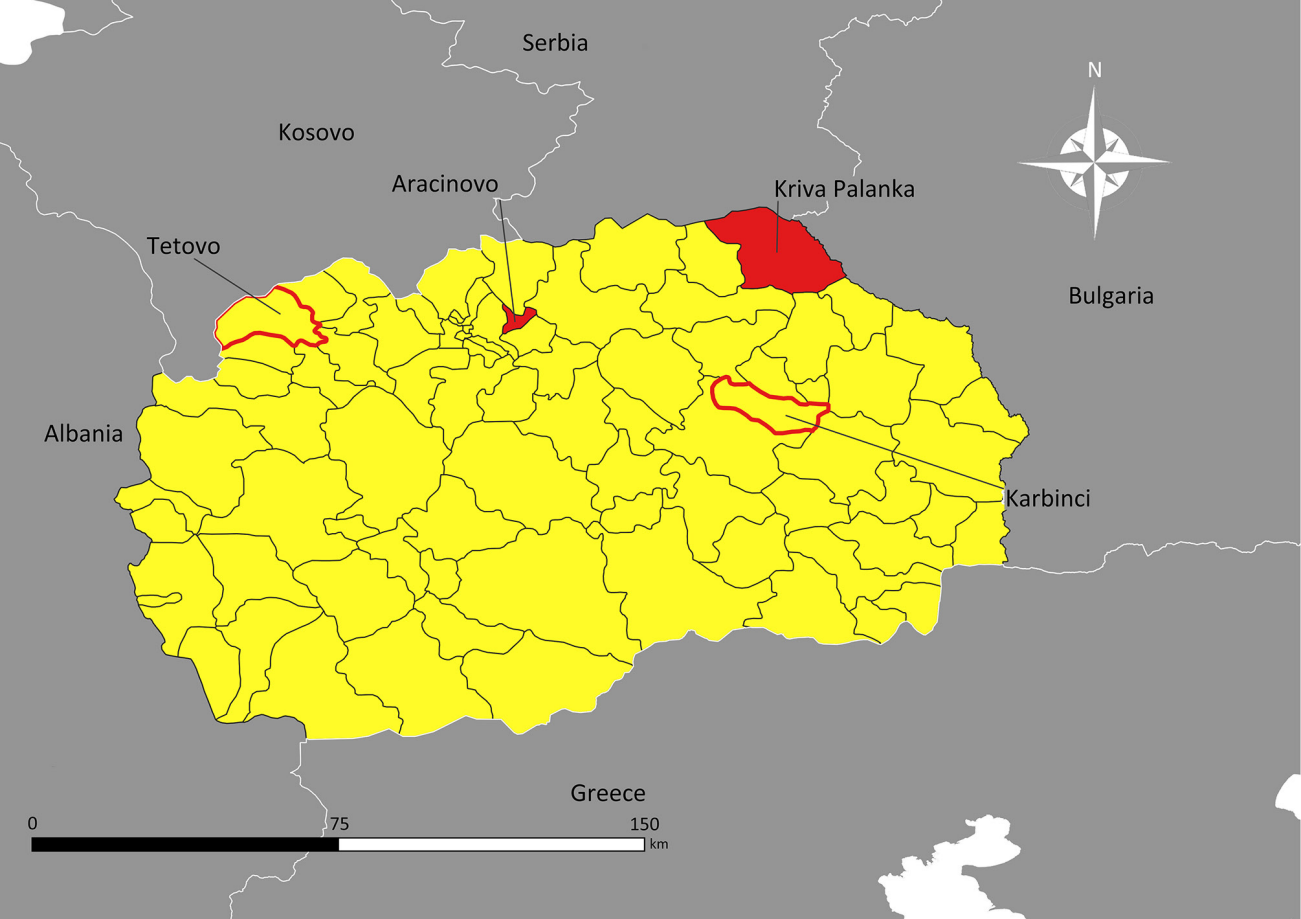
Locations of Crimean-Congo hemorrhagic fever cases in North Macedonia. Red shading indicates municipalities where cases emerged in April–May 2024; red outlines indicate municipalities where previous cases occurred (cases of 1970 and 2023). Shapefile for mapping North Macedonia at district and municipality levels available through GADM Database of Global Administrative Areas version 2.8 (https://gadm.org). Map generated by using QGIS version 3.12 (https://www.qgis.org).

**Figure 2:**
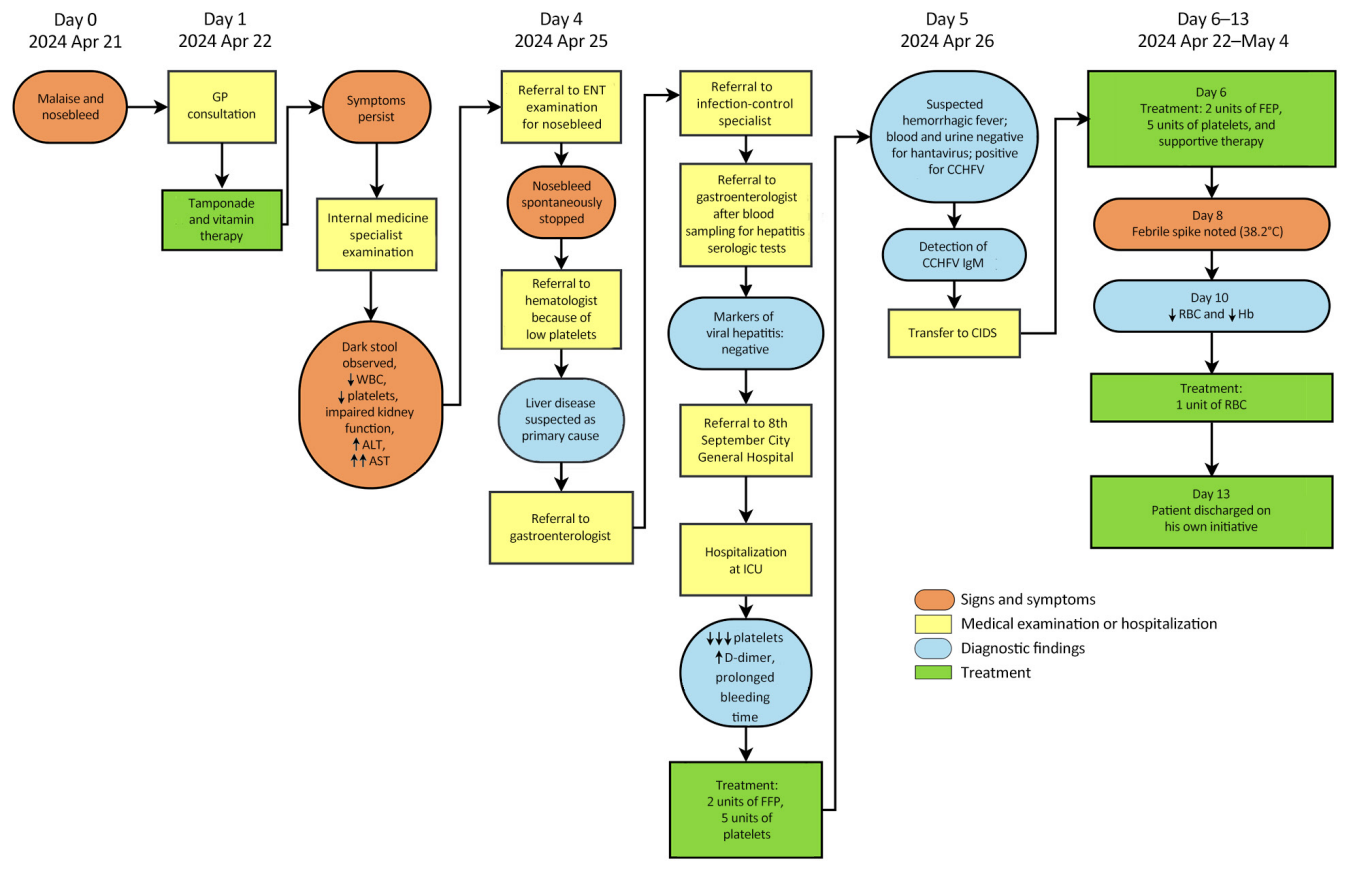
Clinical course, diagnostic findings, treatment, and outcome for CCHF case-patient 1 during CCHF outbreak, North Macedonia, April–May 2024. Up arrows (↑) indicate that a parameter increased and down arrows (↓) that a parameter decreased; multiple up or down arrows indicate degree of increase or decrease. Timeline generated by using open-source software draw.io (https://app.diagrams.net). ALT, alanine transaminase; AST, aspartate aminotransferase; CCHF, Crimean-Congo hemorrhagic fever; CCHFV, CCHF virus; CIDS, Clinic for Infectious Diseases in Skopje; ENT, ear, nose, and throat specialist; FFP, fresh frozen plasma; GP, general practitioner; Hb, hemoglobin; ICU, intensive care unit; RBC, red blood cells (erythrocytes); WBC, white blood cells (leukocytes).

On April 26, 2024, a man in his 60s (case-patient 1) with no notable medical history was admitted to the Clinic for Infectious Diseases in Skopje (CIDS), Skopje, North Macedonia. He resided alone in a rural village in the northeastern region of North Macedonia in the municipality of Kriva Palanka (Figure 1). He worked as a self-employed herder and had not traveled outside the region in the preceding month. On April 14, 2024, the patient noticed a tick attached on his left lower leg and removed it with tweezers. The exposure site was ≈17 km south of the border with Serbia and ≈15 km east of the border with Bulgaria (Figure 1). Seven days after tick removal (day 0), the patient had malaise and persistent nosebleeds, which continued despite nasal tamponade. On day 4, he noticed dark stools, prompting a visit to an internal medicine specialist. Laboratory tests revealed leucopenia, thrombocytopenia, and elevated aminotransferase levels (Appendix 1). The patient was evaluated by multiple specialists and admitted to the intensive care unit at 8th September City General Hospital in Skopje. On day 5, we detected CCHFV in blood and urine samples by using Viasure reverse transcription PCR (RT-PCR) (Certest Biotec, https://www.certest.es), which had a sensitivity of >10 RNA copies/reaction. Upon confirmation of CCHF, the patient was transferred to CIDS for further treatment (Figure 2). We have detailed his clinical course, diagnostic findings, treatment, and outcome (Figure 2).

**Figure 3:**
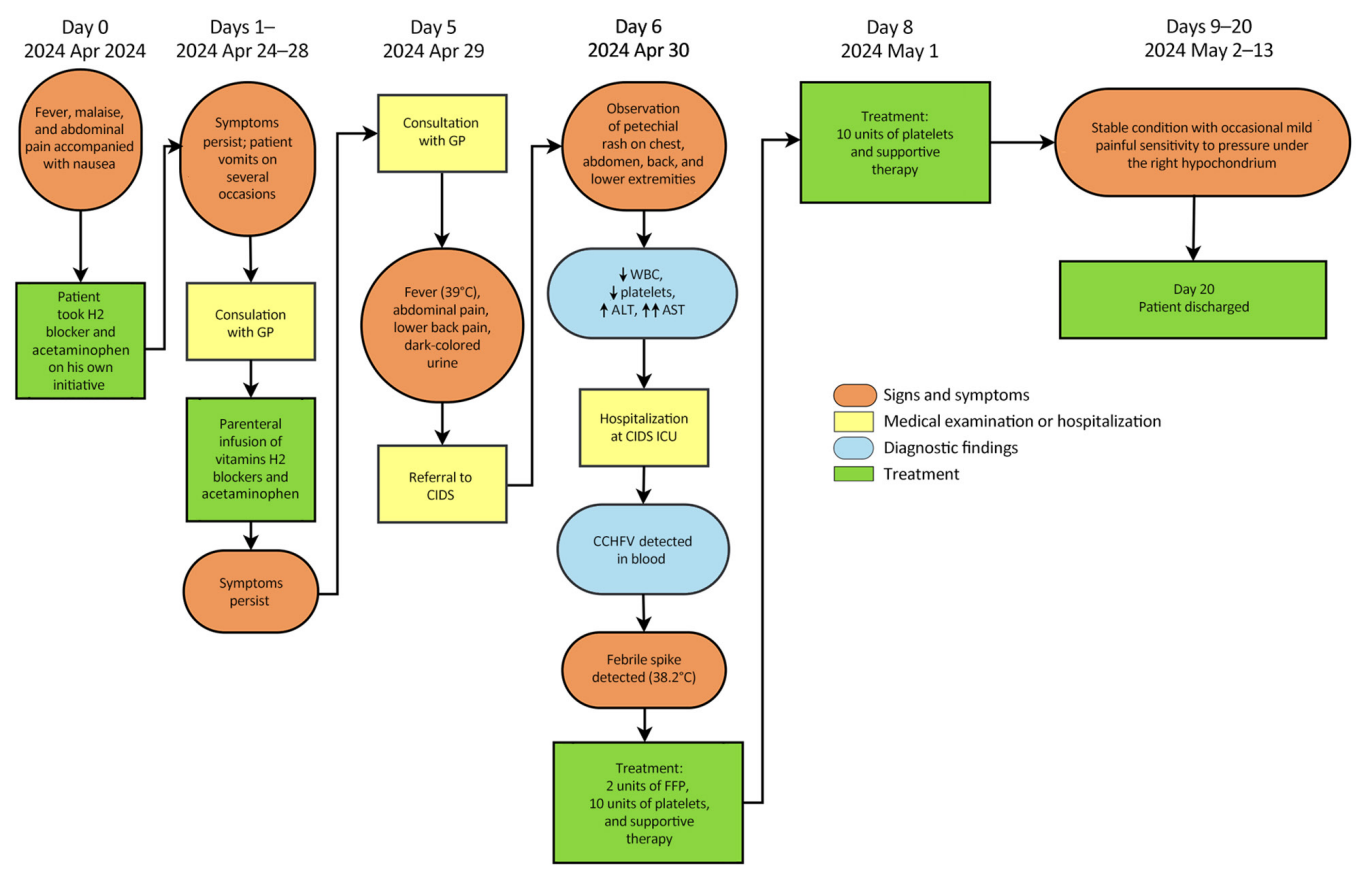
Clinical course, diagnostic findings, treatment, and outcome for CCHF case-patient 2 during CCHF outbreak, North Macedonia, April–May 2024. Up arrows (↑) indicate that a parameter increased and down arrows (↓) that a parameter decreased; multiple up or down arrows indicate degree of increase or decrease. Timeline generated by using opensource software draw.io (https://app.diagrams.net). ALT, alanine transaminase; AST, aspartate aminotransferase; CCHF, Crimean-Congo hemorrhagic fever; CCHFV, CCHF virus; CIDS, Clinic for Infectious Diseases in Skopje; ENT, ear, nose, and throat specialist; FFP, fresh frozen plasma; GP, general practitioner; ICU, intensive care unit; WBC, white blood cells (leukocytes).

On April 30, 2024, a man in his 30s (case-patient 2) with no notable medical history was admitted to CIDS. On April 21, after a local hike, he had noticed a tick attached to his right ankle and removed it with his hands. The patient resided in a rural area of the Skopje Region in the municipality of Arachinovo (Figure 1) and had not traveled outside the area in the preceding month. Three days after tick removal (day 0), he experienced the sudden onset of fever, malaise, and abdominal pain accompanied by nausea (Figure 3). On day 1, he experienced 2 episodes of vomiting and consulted his family physician, who administered parenteral vitamin infusion, H2 blockers, and paracetamol. On day 5, because of persistent symptoms, the patient was referred to CIDS (Figure 3). Upon evaluation at CIDS on day 6, he had a temperature of 39°C and petechial rash distributed on his chest, abdomen, back, and lower extremities. Laboratory findings revealed leucopenia, severe thrombocytopenia, and elevated aminotransferases (Appendix 1). We confirmed CCHFV infection through analysis of blood using Viasure RT-PCR, which had a sensitivity of >10 RNA copies/reaction, as previously described. The patient was hospitalized. We have detailed his clinical course, diagnostic findings, treatment, and outcome (Figure 3).

To characterize the CCHFV strains from the 2 patients, we conducted molecular analysis of blood samples from both patients, followed by phylogenetic analysis of all 3 viral segments. In brief, we collected blood samples on day 5 (case-patient 1) and day 6 (case-patient 2) after symptom onset. After heat inactivation, we extracted nucleic acids and confirmed the presence of CCHFV RNA through RT-PCR as described by Atkinson et al (*5*). Cycle threshold (Ct) values were 35 for case-patient 1 and 37 for case-patient 2, indicating positive results. To further analyze the viral genome, we performed amplicon-based sequencing targeting the Europe-1 genotype on the Oxford Nanopore platform (*6*). We conducted sequence processing and assembly by using Geneious Prime 2024.0.5 (https://www.geneious.com). For case-patient 1, we obtained complete sequences of the small and medium segments and a partial sequence of the large segment. For case-patient 2, we generated only partial sequences of all 3 segments (GenBank accession nos. PQ031235–40). We reconstructed phylogenetic trees by using the IQ-TREE software with the maximum-likelihood method and the general time-reversible plus empirical base frequencies plus invariable site plus discrete gamma 4 substitution model, supported by 1,000 bootstrap replicates for robust statistical inference (*7*). We visualized and edited the trees by using the iTol online tool (*8*).

Phylogenetic analysis revealed the viral isolates from both patients clustered with regional strains within the Europe-1 lineage (genotype V). Of note, the medium segment from case-patient 1 showed high similarity to sequences from Turkey, suggesting a possible reassortment event. To investigate further, we conducted recombination analysis by concatenating genomic segments and aligning them with 5 selected CCHFV genomes using the MUSCLE plugin in Geneious Prime 2024.0.5. We trimmed the alignment to equal lengths and excluded regions containing gaps. We conducted recombination analysis by using RDP4 software (https://rdp4.software.informer.com) with 90% cutoff value for tree permutations, a 500-bp window size, and Bonferroni correction for a p value threshold of 0.05. Bootscan analysis confirmed the reassortment event associated with the medium segment of the virus from case-patient 1 (GenBank accession no. PQ031235.1) (Appendix 2). This evidence strongly supports the hypothesis that the virus infecting case-patient 1 underwent reassortment, probably involving gene flow between CCHFV isolates from Turkey and Kosovo within the Europe-1 lineage.

To evaluate the immune response in both cases, we analyzed serum samples for the presence of CCHFV IgM and IgG by using VectoCrimean-CHF-IgM ELISA and VectoCrimean-CHF-IgG ELISA (VectorBEST, https://en.vector-best.ru), following the manufacturer’s protocols. Both patients exhibited strong IgM reactivity against CCHFV, indicating acute infection. In contrast, CCHFV IgG was undetectable in both cases, consistent with a primary immune response during the early stages of infection.

## Conclusions

Our detailed case studies illustrate the clinical variability and severity of CCHFV infections. Both patients had hallmark symptoms, such as fever, malaise, and thrombocytopenia, but their clinical courses varied considerably in progression and intensity. This study emphasizes the importance of coordinated efforts by the Balkan Association for Vector-Borne Diseases in managing and mitigating the effect of zoonotic diseases in the region. The reemergence of CCHF in North Macedonia, which had 5 cases reported in 2023 and 2024 from 4 distinct regions, underscores the necessity for sustained surveillance of all components of the infection chain, including ticks, wildlife, domestic animals, and humans. Rapid diagnostic tools and comprehensive public health strategies are critical in preventing and controlling future outbreaks. Of note, the identification of possible reassortment events involving CCHFV isolates from Turkey and Kosovo within the Europe-1 lineage highlights the importance of North Macedonia as a geographic interaction zone for multiple viral lineages. These findings emphasize the need for further research into ecology and evolution of the virus in this region to better assess the risks to human and animal health.

Appendix 1 Routine laboratory test results for 2 case-patients infected with diverse Europe-1 Crimean-Congo hemorrhagic fever virus strains, North Macedonia, 2024.

Appendix 2 Bootscan and phylogenetic analysis of Crimean-Congo hemorrhagic fever virus isolates, North Macedonia, April–May 2024.
