# Supplementary material for: Two Human Infections with Diverse Europe-1 Crimean-Congo Hemorrhagic Fever Virus Strains, North Macedonia, 2024
Source: Emerg Infect Dis. 2025 Feb;31(2):313–7. doi: 10.3201/eid3102.241249 (PMC11845147; doi:10.3201/eid3102.241249)
Supplement: Appendix 2 — Bootscan and phylogenetic analysis of Crimean-Congo hemorrhagic fever virus isolates, North Macedonia, April–May 2024. [file 24-1249-Techapp-s2.pdf]

Article DOI: https://doi.org/10.3201/eid3102.241249

EID cannot ensure accessibility for supplementary materials supplied by authors. Readers who have difficulty accessing supplementary content should contact the authors for assistance.

Two Human Infections with Diverse Europe-1 Crimean-Congo Hemorrhagic Fever Virus Strains, North Macedonia, 2024

Appendix 2

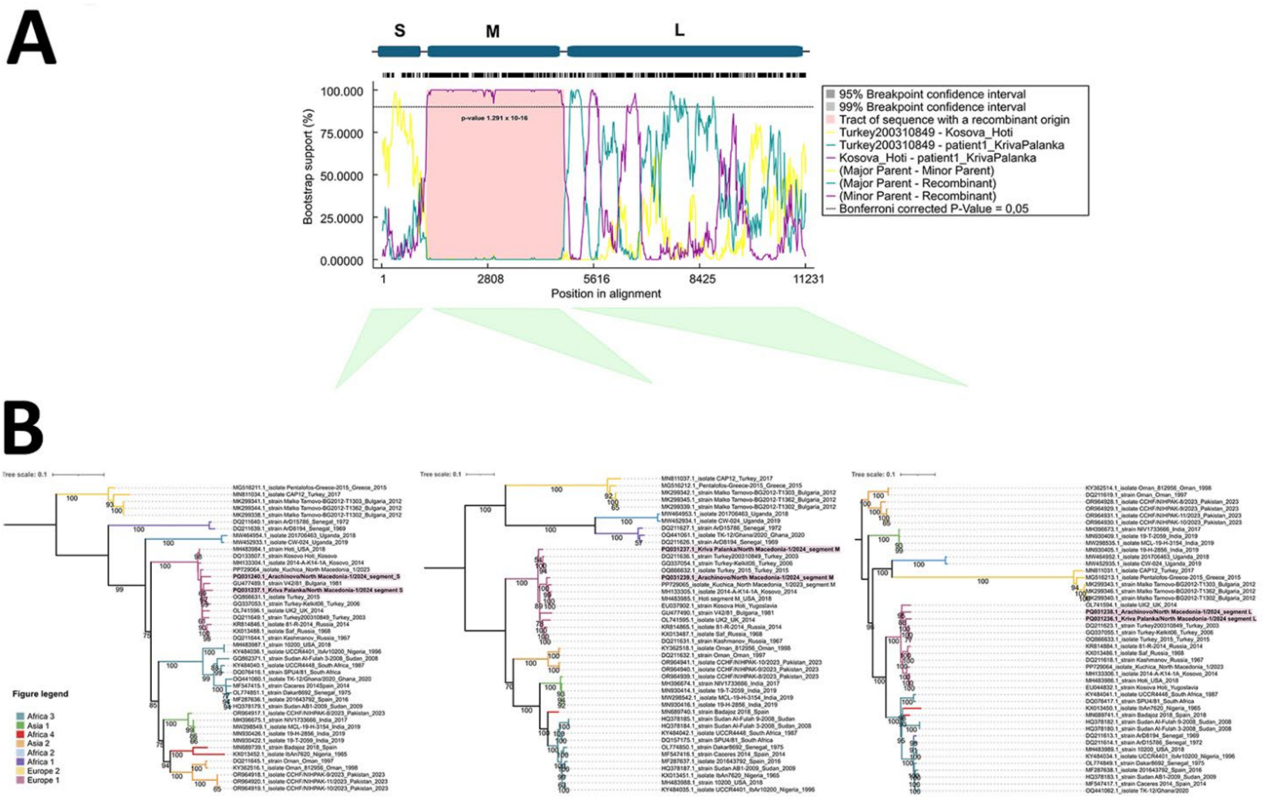

Appendix 2 Figure. Bootscan and phylogenetic analysis of Crimean–Congo hemorrhagic fever virus isolates, North Macedonia, April–May 2024. A) Bootscan analysis of the CCHFV isolate Arachinovo/North Macedonia-1/2024 (GenBank accession nos. PQ031238.1–PQ031240.1) and Kriva Palanka/North Macedonia-1/2024 (GenBank accession nos. PQ031235.1–PQ031237.1). Approximate positions of the genes are also depicted above the Bootscan plot. B) Maximum-likelihood phylogenetic trees of the 3 viral genomic segments. Sequences examined in this study are highlighted with colored background. L, large; M, medium; S, small.
